# Clinical application of metagenomic next-generation sequencing in tuberculosis diagnosis

**DOI:** 10.3389/fcimb.2022.984753

**Published:** 2023-03-28

**Authors:** Ying Liu, Huifen Wang, Yaoguang Li, Zujiang Yu

**Affiliations:** ^1^Gene Hospital of Henan Province, The First Affiliated Hospital of Zhengzhou University, Zhengzhou, China; ^2^Department of Infectious Diseases, The First Affiliated Hospital of Zhengzhou University, Zhengzhou, China

**Keywords:** tuberculosis, metagenomic next-generation sequencing, clinical diagnosis, traditional detection method, diagnosis performance

## Abstract

**Objective:**

The purpose of this study was to evaluate the clinical diagnostic value of metagenomic next-generation sequencing (mNGS) for tuberculosis (TB).

**Methods:**

This retrospective study included 52 patients with suspected TB infection. mNGS, targeted PCR, acid-fast staining and, T-SPOT.TB assay were performed on the specimen. The positive rate of mNGS and traditional detection methods was statistically analyzed. Pathological tests were performed when necessary.

**Results:**

In total, 52 patients with suspected of TB in this study were included in the analysis, and 31 patients were finally diagnosed with TB. Among 52 patients, 14 (26.9%) cases were positive for acid-fast staining. The positive rate of T-SPOT.TB assay in 52 patients was 73.1% (38/52). Among 52 patients, 39 (75%) were detected positive for *Mycobacterium tuberculosis* (*M*TB) by mNGS. Regarding the detection rate of MTB, mNGS were as high as 75% (39/52), whereas acid-resistant staining was only 26.9% (14/52), which showed a statistically significant difference (p<0.05). The positive rates of T-SPOT.TB assay and mNGS were not statistically significant (p>0.05). Of the 52 suspected TB patients, 24 had targeted PCR, of which 18 were PCR positive. In 24 patients, the positive rate of PCR was 75%, and the positive rate of mNGS was 100%, with statistical difference between them (p<0.05).

**Conclusions:**

The detection rate of MTB by mNGS was higher than that by conventional acid-fast staining and PCR, but not statistically significant compared with T-SPOT.TB assay. As an adjunctive diagnostic technology, mNGS can be combined with traditional detection methods to play a guiding role in the diagnosis and treatment of TB.

## Introduction

TB remains one of the infectious diseases with high incidence and mortality worldwide, with about 10 million new TB cases worldwide each year, of which 1 million are children. In the World Health Organization’s global progress report on eliminating TB, the death rate from TB is the highest of any infectious disease, surpassing that of AIDS. New and recurrent cases are on the rise, with drug-resistant TB patients making up the main part of the new cases. TB control remains a huge challenge (Furin et al., 2019; Reuter et al., 2019; Suárez et al., 2019; Harding, 2020; MacNeil et al., 2020).The common diagnostic methods of TB include tuberculin skin tests, T-SPOT.TB assay, acid-fast staining, mycobacterial culture, molecular diagnosis, where molecular diagnostics include PCR, Xpert MTB/RIF. The tuberculin skin test can produce false positives in people vaccinated with Bacille Calmette-Guerin (BCG) or infected with *nontuberculous mycobacteria* (NTB), and sensitivity is very low in people with immune deficiency (Acharya et al., 2020). T-SPOT.TB assay, as a commonly used method to assist the diagnosis of TB, has better sensitively and specificity than culture method, but is also susceptible to some factors, such as older patients, overweight and longer hospitalized patients prone to false negative and cannot distinguish between active and latent infected TB (Pan et al., 2015; Luo et al., 2021). Although the acid-fast staining is quick and effective in diagnosing TB, it has a false negative rate of 30%-65%, which makes TB often missed. However, the culture method is time-consuming, usually taking 2to 8 weeks to produce results, so it is not useful for clinical guidance (Campos et al., 2016; Khadka et al., 2019).The new molecular diagnostic technique Xpert MTB/RIF can detect MTB and its resistance to rifampicin in less than 2 hours, improving TB diagnosis. However, Xpert MTB/RIF could not identify new resistant mutations and could not distinguish whether the mutations led to changes in the amino acid sequence (Opota et al., 2019; Acharya et al., 2020).

Less than 1% of all microorganisms in the environment are culturable, and some are not culturable. Metagenomes hold the promise of providing information on all microbiota, especially for previously unrecognized and unculturable microorganisms (Borroni et al., 2019). The Human metagenomic Project mainly covers the skin, gastrointestinal tract, urogenital tract, oral and nasal mucosa, but there is also an emerging field of research - ophthalmology (Gallon et al., 2019; Parekh et al., 2019).Clinical mNGS has been widely used in the diagnosis of infectious diseases by providing comprehensive and rapid analysis of the genetic material of the microbe and host in patient samples. mNGS can be used for routine identification, genotyping and virulence detection of pathogenic microorganisms (Gautam et al., 2018; Guthrie et al., 2018; Chiu and Miller, 2019). Many studies have demonstrated the value of mNGS in diagnosing pathogenic microorganisms and guiding faster and better antimicrobial therapy, including central nervous system infections, lung infections, bloodstream infections, eye disease, infections in immunocompromised patients and so on (Wilson et al., 2018; Crawford et al., 2020; Shi et al., 2020; Thakur, 2020; Borroni, 2021a; Borroni, 2021b; Gu et al., 2021). The diagnosis of TB faces great challenges, but most of the studies on the diagnostic role of mNGS in TB are case reports, and there are few clinical studies. Therefore, we conducted a retrospective study to evaluate the diagnostic value of mNGS in TB.

## Methods

### Study design and participants

We included suspected TB patients admitted to Henan Gene Hospital, The First Affiliated Hospital of Zhengzhou University, from January, 2020 to March 31, 2021. We conducted a retrospective study to evaluate the role of mNGS in the diagnosis of TB. Samples from all patients were tested for mNGS, T-SPOT.TB assay and acid-fast staining, and some patients underwent pathological examination and targeted PCR. We counted the patient’s baseline information, including patient age, sex, highest body temperature on the day of admission, whole blood cell count, specimen types, specimen smear, pathological results, etc.

Suspected TB included met the following criteria (include at least one of the following): 1) TB exposure history. 2) Symptoms of TB poisoning such as cough, fever, night sweats or weight loss. Extrapulmonary TB has corresponding symptoms. 3) Imaging features of tuberculosis. The final clinical diagnostic criteria for tuberculosis include: 1) positive for pathogens (including acid-fast staining smears or specimen cultures). 2) Imaging and clinical manifestations excluded other diseases and histopathological confirmed tuberculosis foci. 3) After 3 months of anti-tuberculosis treatment, the tuberculosis changes abated or disappeared.

We have established the mNGS Professional Committee consisting of expert groups. This professional evaluation team evaluated our cases, and the existence of the expert team ensured the reliability of our original data and the correctness of our clinical diagnosis. The final clinical diagnosis of suspected TB, including TB and extrapulmonary TB, will be made after the determination of the expert panel.

### Sample preparation, DNA extraction, library construction and sequencing

BALF, biopsy tissue, CSF, blood, pus, lymph node puncture, sputum, and joint fluid samples were collected from patients according to the standard clinical procedure. Blood samples were centrifuged at 4°C at 1500 rpm for 10 minutes. Then the plasma layer was carefully collected and transferred to a new tube and centrifuged at 4°C at 12000 rpm for 10 minutes. Cell-free DNA (cfDNA) from plasma samples was extracted by using the TIANamp Micro DNA DP316 Kit (Tiangen Biotech, Beijing, China) following the manufacturer’s protocol. DNA from other samples was extracted using the QIAamp^®^ UCP Pathogen Kit (Qiagen, Germany) according to the manufacturer’s recommendations. The extracted cfDNA and DNA samples were quantified with a Qubit Fluorometer (Thermo Fisher Scientific, CA, USA).

The extracted cfDNA samples were used to construct DNA libraries by using the VAHTS Universal DNA Library Prep Kit V3 for Illumina^®^ (Vazyme, Nanjing, China). For other samples, DNA libraries were prepared by using the TruePrep DNA Library Prep Kit V2 for Illumina^®^ (Vazyme, Nanjing, China) according to the manufacturer’s manuals. The Agilent 2100 Bioanalyzer (Agilent Technologies, Santa Clara, USA) was used for library quality control. All libraries were pooled with other libraries by using different index sequences and sequenced on an Illumina NextSeq 550Dx platform with the single end 75bp sequencing option. For each run, no template control (NTC) samples (Nuclease-free H_2_O) were also pooled to monitor reagent and laboratory background.

### Bioinformatics analysis

Fastq-format data were generated for each sample by using bcl2fastq software (v2.20.0.422, parameters used: –barcode-mismatches 0 –minimum-trimmed-read-length 50). Adapt sequences and low-quality reads were removed using cutadapt v2.10 (-q 25, 25 -m 50). The remaining high-quality reads were first depleted for human sequences by mapping to the human genome (hg38, https://hgdownload.soe.ucsc.edu/downloads.html#human) using bwa-mem 2 v2.1 with default parameters, all unmapped reads were then aligned to the NCBI nt database (https://ftp.ncbi.nlm.nih.gov/genomes/) by using BLAST v2.9.0+ (-task megablast -num_alignments 10 -max_hsps 1 -evalue 1e-10). Alignments were required to be full-length with an identity of at least 95%. A customized Python script was used to identify species-specific alignments. Only the alignments that fulfill the above-mentioned criteria were used for further pathogen identification. The remaining microorganisms were defined as credible if the following criteria were met: (1) the microbe had at least 2 non-redundant, mapped reads per million (RPM) raw sequence reads (except for the *Mycobacterium tuberculosis*), and the microbe was not detected in corresponding NTC sample or the RPM(sample)/RPM(NTC) ≥ 5, which was our empirical cutoff for differentiating true-positive from background contamination, (2) for *Mycobacterium tuberculosis complex*(MTBC), due to the difficulty of detection, when at least one taxon-specific, high-quality aligned read were identified, the sample were reported as MTBC positive.

### Statistical analysis

All data were analyzed by SPSS software (release26.0). Continuous data are considered nonparametric. Continuous variables were expressed as median and interquartile range (IQR), and the Shapiro-Wilk test (p<0.05) was used for continuous variables that were not normally distributed. The classified data were represented by case number (n) and percentage (%). Chi-square test (McNemar test) was used for comparison. P <0.05 was statistically significant without multiple testing adjustment.

## Results

### Clinical Characteristics of the patients

A total of 52 suspected TB patients and 53 specimens were collected for statistical analysis. The baseline information for the patients was shown in **Table 1**. The median age of patients was 50.0 years (IQR,34.8;66.0), 51.9% (27/52) of whom were male. The median number of hospital days for the 52 patients was 11 (IQR,5;16), and the median hospital cost was $2659.23(IQR, 281;6161) (**Table 1**). When discharged from hospital, 39 (75%) improved,7(13.5%) cured and 2 (3.8%) with poor efficacy (**Table 1**).53 specimens were sent for mNGS, including 16(30.2%) of alveolar fluid (BALF), 25 (47.2%) of biopsy tissue, 5 (9.4%) of cerebrospinal fluid (CSF),3 (5.7%) of blood,1 (1.9%) of pus, 1(1.9%) of lymph node puncture, 1 (1.9%) of sputum, and 1(1.9%) of joint fluid (**Table 2**). The 31 mNGS TB positive patients were consistent with the clinical diagnosis, including 20 cases of pulmonary TB, 2 cases endobronchial TB,4 cases of tuberculous meningitis,1 case of chest wall tissue infection, 1 case of mediastinal lymph node infection, 2 cases of joint TB, and 1 case of tuberculous pleurisy. The remaining 8 mNGS positive cases had insufficient evidence to determine tuberculosis infection.

**Table 1:** Baseline characteristics of the study population.

Characteristic	Clinical value	P-value
Age (years), Median (IQR)	50.0 (34.8;66.0)	<0.05
Hospital stays (day), Median (IQR)	8 (5.0;16.0)	<0.05
Sex		
Male, N (%)	27 (51.9)	
Female, N (%)	25 (48.1)	
WBC (10^9^/L)	6.8 (4.60;7.90)	<0.05
Body temperature (°C), Median (IQR)	36.8 (36.5;37.2)	<0.05
Curative effect		
Cure, N (%)	7 (13.5)	
Improve, N (%)	39 (75)	
Uncured, N (%)	2 (3.8)	
Death, N (%)	0	
Other, N (%)	4 (7.7)	
Hospitalization costs (dollar), Median (IQR)	2659.23 (281;6161)	<0.05

The Shapiro-Wilk test was used for continuous variables that were not normally distributed. P< 0.05 was considered a non-normal continuous variable.

IQR, interquartile range; WBC, white blood cell count.

**Table 2:** Sample type.

Sample, N (%)	
BALF Biopsy tissue CSF Blood Pus Synovial fluid Lymph node puncture fluid sputum	16 (30.2) 25 (47.2) 5 (9.4) 3 (5.7) 1 (1.9) 1 (1.9) 1 (1.9) 1 (1.9)

CSF, cerebrospinal fluid; BALF, bronchoalveolar lavage fluid.

### Diagnostic performance of mNGS and targeted PCR

We randomly selected 24 samples from 52 patients for PCR to validate the mNGS results. Of the 24 patients who underwent PCR, 18(18/24,75%) specimens were positive. Of the 24 patients tested by mNGS, 24 (24/24,100%) were positive. In 24 patients, the positive rate was 75% for PCR and 100% for mNGS, indicating statistical significance between the two assays (100% vs 75%; P =0.009). In 24 patients, mNGS and clinical diagnosis were consistent in 19 cases (79.2%), PCR and clinical diagnosis were consistent in 17 cases (70.8%), and there was no statistical significance between them (p=0.505).

### Comparative analysis of mNGS and acid-fast staining pathogen detection rate

**Figure 1:**
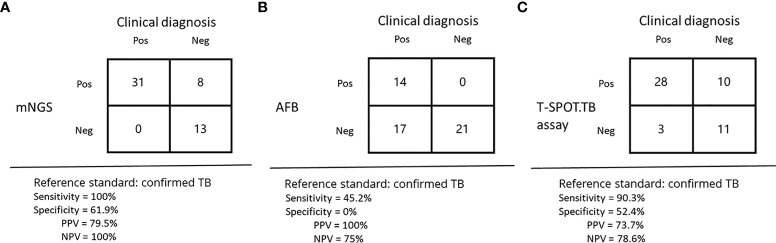
Diagnostic results of mNGS, acid-fast staining, and the T-SPOT.TB assay in suspected TB. **(A–C)** performance of different methods for diagnosis of tuberculous.

**Figure 2:**
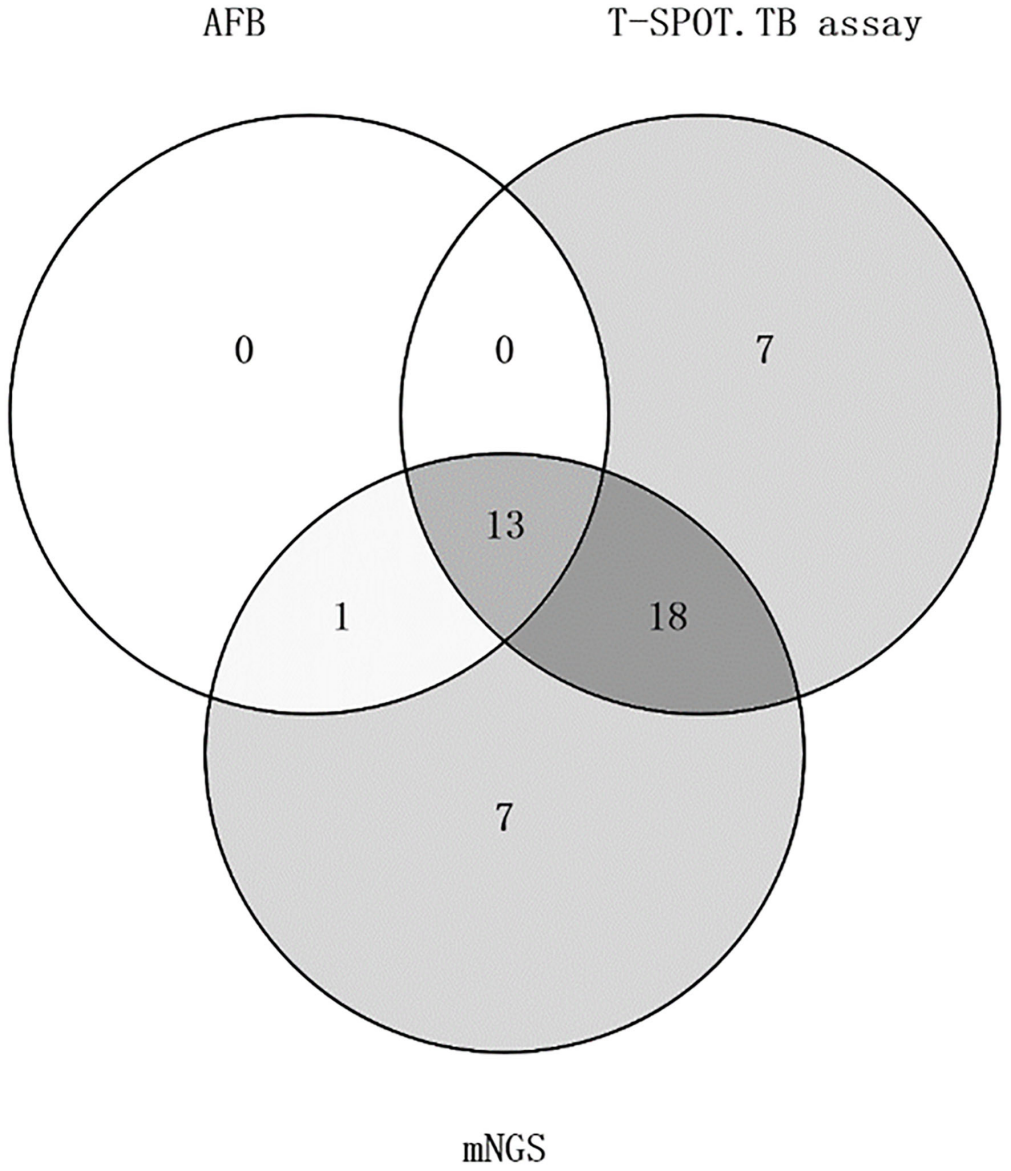
Venn diagram of patients with mNGS, acid-fast staining, and the t-spot. TB assay positive staining.

In the detection rate of MTB, mNGS detection rate was as high as 75% (39/52), while acid-fast staining only 26.9% (14/52), the difference was statistically significant (P<0.05). Acid-fast staining, mNGS and T-SPOT were positive in 25% (13/52) of the patients. One patient was positive for acid-fast staining and mNGS but negative for T-SPOT (**Figure 2**). With clinically confirmed tuberculosis as the reference standard, mNGS had clinical sensitivity of 100%, clinical specificity of 61.9%, positive predictive value of 79.5%, and negative predictive of 100% (**Figure 1A**). Similarly, the clinical sensitivity of acid-fast staining was 45.2%, the specificity was 0, the positive predictive value was 100%, and the negative predictive value was 75% (**Figure 1B**).

### Comparative analysis of mNGS and T-SPOT.TB assay pathogen detection rate

Among all cases, 38 cases were detected by T-SPOT.TB, with a positive rate of 73.1%. Although 39 cases were detected by mNGS, a positive rate of 75%, the detection rates of mNGS and T-SPOT were not statistically different. The percentage of patients with positive T-SPOT and mNGS but negative acid-fast staining was 34.6% (18/52). T-SPOT.TB assay alone detected 13.5% (7/52) of MTB, the same as mNGS. In contrast to mNGS and T-SPOT, acid-fast staining did not detect MTB alone (**Figure 2**). With clinically confirmed tuberculosis as the reference standard, T-SPOT.TB assay had clinical sensitivity of 90.3%, clinical specificity of 52.4%, positive predictive value of 73.7%, and negative predictive of 78.6% (**Figure 1C**).

### Diagnostic performance of mNGS, acid-fast staining, and T-SPOT.TB assay in confirmed TB

Of the 52 patients, 31 were eventually diagnosed with TB. Of the 31 patients with confirmed TB, 31 were mNGS positive, 14 were acid-fast staining positive, and 28 were T-SPOT.TB assay positive. Among the 31 patients with confirmed TB, the positive rate of mNGS was 100% (31/31), acid-fast staining was 45.2% (14/31), and T-SPOT.TB assay was 90.3% (28/31). There were 13 TB patients with positive mNGS, acid-fast staining and T-SPOT.TB assay. In other words, of the 14 patients with positive acid-fast staining and mNGS, one patient had a negative T-SOPT.TB assay, and the remaining 13 patients had positive mNGS and T-SPOT.TB assay. In these 31 patients, the positive rates of mNGS and acid-fast staining were statistically significant (p<0.05), while the positive rates of mNGS and T-SPOT were not (p>0.05).

Of the 31 confirmed TB cases, 20 were pulmonary TB and 11 were extrapulmonary TB. In 20 cases of TB, the positive rate of mNGS was 100% (20/20), while that of T-SPOT.TB assay was 95% (19/20). The positive rate of acid-fast staining was only 50% (10/20), which was significantly lower than mNGS and T-SPOT.TB assay. In 20 cases of pulmonary TB, the positive rates of mNGS and acid-fast staining were statistically significant(p<0.05). In 11 cases of extrapulmonary TB, the positive rate of mNGS was 100% (11/11). In all extrapulmonary TB, the positive rates of acid-fast staining and T-SPOT were36.4% (4/11) and 81.8% (3/11) respectively. mNGS were statistically significant with acid-fast staining and T-SPOT.TB assay in extrapulmonary TB.

## Discussion

TB is a major public health problem worldwide, placing a heavy economic burden on people around the world, especially in poor areas. The high treatment cost of TB makes most TB families heavily in debt, especially multidrug-resistant TB longer hospitalization time, longer treatment time, aggravating poverty. Early timely and correct diagnosis of TB is a necessary condition to cure TB, especially latent TB infection. Although case studies demonstrate the great potential of mNGS in diagnose disseminated TB and extrapulmonary TB, there are few studies of overall TB (Zhang et al., 2019; Ye et al., 2021). This cohort study applied mNGS to clinical samples to assess its diagnostic efficacy for TB and extrapulmonary TB. We chose the use of clinical diagnosis rather than conventional testing as a comparison because of the limitation of routine methods to extrapulmonary specimens, which are the majority of extrapulmonary TB specimens in our study. Despite the lack of culture for clinical diagnosis, pathologic results and follow-up greatly reduced the possibility of misdiagnosis.

The smear method is a relatively simple, inexpensive and convenient procedure that is suitable for a variety of hospitals. However, the level of hospital equipment and the level of inspectors is not consistent, so the diagnostic value of smear method cannot be standardized, the difference between hospitals is very significant. Acid-fast staining relies on the high number of bacteria present in the sputum to give accurate results. Several studies have shown that the sensitivity of acid-fast staining can be as low as 8% or as high as 55.68% (Qiu et al., 2022; Stadelman et al., 2022; Uddin et al., 2022; Zhang et al., 2022). Although mNGS is slightly more sensitive than T-SPOT.TB assay, for TB, a trade-off from a cost/time perspective would be beneficial. In China, the MNGS test costs about $450 per sample, while T-SOPT.TB assay costs about $73.05 to $100. The detection period for mNGS is one day, while the detection time for T-SPOT.TB assay is about 12 hours. However, studies have shown that mNGS can detect drug-resistant genes or detect mixed pathogens, changing the clinical treatment of TB patients and reduce the cost effectiveness.

In the diagnostic efficacy of TB, mNGS is better than anti-acid staining, even in some patients with negative pathological TB-DNA tests. Especially in tuberculous meningitis, all five patients were negative for acid-fast staining but positive for mNGS. This suggests that mNGS plays a significant role in the diagnosis of tuberculous meningitis. In a retrospective study, mNGS was significantly more effective in diagnosing tuberculous meningitis than acid-fast staining, PCR, and culture. Studies have shown that mNGS can be recommended as a first-line test for tuberculous meningitis (Wang et al., 2019). As the most widely used method of diagnosis TB, acid-fast staining has the advantages of fast, convenience and low cost, but its detection rate is low and easy to cause missed diagnosis of TB. According to the literature, the diagnosis of extra-pulmonary TB is very difficult. This study demonstrates great potential for diagnosis of extrapulmonary TB. For areas with a high incidence of TB, the application value of mNGS is very valuable for smear-negative TB. Conventional PCR is restricted to gene sequences of known pathogenic microorganisms, and mNGS can sequenced the whole microbial genome in the sample, especially for emerging resistance genes, etc. (Sun et al., 2021).In addition, our study showed the advantage of mNGS meeting mixed infections with multiple pathogens. One case of *Klebsiella pneumoniae* and *Candida albicans* detected by mNGS was consistent with blood culture results. mNGS detected not only *M. tuberculosis* but also different types of viruses in the samples from the four patients. All of these indicate the potential of mNGS for the diagnosis of mixed infections.

## Limitations

Because MTB is an intracellular bacterium, this may result in reduced susceptibility to mNGS. But mNGS can detect mutations in drug-resistant genes and type strains. Compared to traditional culture methods, mNGS detection is faster and unbiased, required no prior suspicion of certain pathogens. This retrospective study has some drawbacks. Firstly, the sample size we include was relatively small, only 52 cases. Prospective studies with a larger sample size are needed to further evaluate the diagnostic value of mNGS in TB. Second, the patients we included did not undergo traditional culture, so prospective studies are needed to compare mNGS with traditional detection methods.

## Conclusions

This study demonstrates that mNGS has high value for TB diagnosis, especially for tuberculous meningitis that can be recommended as a first-line diagnostic approach. For smear-negative TB, mNGS showed good diagnostic effects. This is of great significance for the prevention and control of TB.

## Data availability statement

The datasets presented in this study can be found in online repositories. The names of the repository/repositories and accession number(s) can be found below: CNGB, CNP0002261.

## Ethics statement

The studies involving human participants were reviewed and approved by The First Affiliated Hospital of Zhengzhou University (Approval number 2021-KY-0230). Written informed consent to participate in this study was provided by the participants’ legal guardian/next of kin. Written informed consent was obtained from the individual(s), and minor(s)’ legal guardian/next of kin, for the publication of any potentially identifiable images or data included in this article.

## Author contributions

ZY and YLiu analyzed and interpreted data. YLiu and HW wrote the manuscript. HW and YLi revised the article. HW and YLiu collected the data. All the authors agreed to the final manuscript.
